# An In Vitro Procedure for Phenotypic Screening of Growth Parameters and Symbiotic Performances in *Lotus corniculatus* Cultivars Maintained in Different Nutritional Conditions

**DOI:** 10.3390/plants5040040

**Published:** 2016-10-13

**Authors:** Vladimir Totev Valkov, Maurizio Chiurazzi

**Affiliations:** Institute of Biosciences and Bioresources, National Council of Research (CNR), Via P. Castellino 111, 80135 Napoli, Italy; vladimir.valkov@ibbr.cnr.it

**Keywords:** legumes, phosphate use efficiency, symbiotic nitrogen fixation

## Abstract

The establishment of legumes crops with phenotypic traits that favour their persistence and competitiveness in mixed swards is a pressing task in sustainable agriculture. However, to fully exploit the potential benefits of introducing pasture-based grass-legume systems, an increased scientific knowledge of legume agronomy for screening of favourable traits is needed. We exploited a short-cut phenotypic screening as a preliminary step to characterize the growth capacity of three different *Lotus corniculatus* cvs cultivated in different nutritional conditions as well as the evaluation of their nodulation capacities. This experimental scheme, developed for legume species amenable to grow on agar plates conditions, may represent a very preliminary step to achieve phenotypic discrimination on different cultivars.

## 1. Introduction

Legumes are the third largest group of angiosperms and the second largest group of food and feed crops grown on the earth as they are cultivated on 12%–15% of available arable land of the planet and are responsible for more than 25% of the world’s primary crop production [1]. In addition to a primary importance in the human and animal diet, legumes play a crucial role in sustainable agriculture by providing large quantities of soil N through their peculiar endo-symbiotic interaction with soil bacteria of the genus rhizobia leading to reduction of the atmospheric nitrogen into nodule organs. This biological process represents a safe and valuable alternative to the industrial procedure for N reduction [2]. In particular, in the agro-food system, legume-based grazing systems provide substantial advantages when adopted in the intensive ruminant production chain that contribute primarily to a number of environmental problems as this is based on high use of inorganic N-fertilizers on grasslands. These include emissions of N to the environment and pollution of surface and ground water, deterioration of soil structure and fertility, consumption of fossil fuels and greenhouse gas emissions associated with fertilizer manufacture and its distribution and application. Legume-based grazing systems have the ability to reduce environmental problems by increasing the Nitrogen Use Efficiency (NUE) avoiding a high transient surplus of soil mineral N. Ultimately, the potential economic gain for farmers associated with the exploitation of legume and grass–legume silages when compared with grass silages has been estimated at an average of 137 € ha^−1^ [3]. 

Recently, the interest in mixed crops between legumes and grasses has highly increased due to their importance for sustainable and ecologically friendly agriculture [4,5] as legume nitrogen fixation capacity also providing at the same time the grass component in nitrogen [6,7]. In general, the survival of legumes in pastures depends mainly on their competitiveness compared to that of associated grasses. Native ecotypes are, on average, more persistent and better adapted than commercial varieties, because of their high fitness and productivity within the specific environment in which they have been selected [3,8]. Environmental factors, such as temperature, soil pH, nutrient availability, are important in ensuring successful establishment in temperate areas. In particular, soils characterized by low content of inorganic N nutrients such as nitrate and ammonium represent an obvious selective advantage for legumes because of their Symbiotic Nitrogen Fixation (SNF) capacity. SNF is an excellent example of how the plants adapt to the changing environment, as nodulation does not occur when sufficient and readily available amount of fixed nitrogen in the form of nitrate and ammonium is present in the soil. In particular, nitrate dependent inhibitory mechanisms based on both local and systemic signalling regulatory pathways have been largely characterized [9,10,11,12,13,14,15,16,17]. A successful competition between species in a mixture through an appropriate choice of the components, can achieve an improved Nitrogen NUE favouring SNF at the same time. However, a dynamic relationship exists between legume and grass components as for example assimilation of soil N from grasses reduces its suppressing effect on N fixation. On the other hand, as a result of the accumulation of soil N by SNF, grass component dominates and N fixation decreases [18,19]. Competition for soil N in mixtures can have a beneficial effect on the SNF process leading to higher N accumulation in the grass component. Another inorganic nutrient that is often limiting is phosphorus as pasture legumes have a relatively high requirement for this element that must be provided by application of phosphate fertilizer. For this reason, a screening aimed to the identification of legume cultivars with an increased P uptake and utilization capacities possibly combined to an optimal response to low N conditions, has a crucial impact on the sustainability of pastureland. In this context, it is convenient to compare different cultivars originated and selected in different regions within the European area, characterized by a wide range of environmental conditions.

Among legumes, the genus Lotus represent a perfect example of genetic variability with 180 species world wide distributed and adapted to different environmental conditions with only few of them that have been domesticated and selected through plant breeding. Lotus species are normally used to improve the quality of pastures because of their high content of condensed tannins that prevents bloating in ruminants [20,21]. The Mediterranean basin is the area of major distribution of agronomical important Lotus species and *L. corniculatus* is the one with the highest agronomical impact. *L. corniculatus* is used in agriculture practices as forage either at vegetative stage for grazing or as conserved hay or silage and even as potential bio-remediator in contaminated soils because of its intrinsic resilience to high concentrations of metals and metalloids in low fertility soils [22,23]. Lotus genus has acquired great interest also because species such as *L. corniculatus* and *L. japonicus* are amenable to tissue culture [24]. In particular, *L. japonicus* is a diploid, auto-compatible plant, which in 1992 has been proposed as a legume model system [25,26]. Since then, an amazing improvement of the understanding of mechanisms underlying plant-microbe symbiotic interactions has been achieved through the exploitation of *L. japonicus* and many scientific platforms and protocols have been established for investigation of plant growth phenotypes and symbiotic performances. 

We propose a pilot experimental scheme based on germination and growth on agar plate conditions to analyze morphological and quantitative parameters. Although axenic conditions limits the analyses because of the short period of plant growth, artefact effects due to a reduced area for growth and gas exchanges and inability to analyze the dynamic of nutrient uptake, the proposed experimental set up provides strictly homogenous starting conditions either in terms of plant starting material (synchronized seedlings) and nutrient availability (controlled media pH and composition) hence allowing reliable growth rate-based analyses of underground and aerial phenotypic parameters within a short period of time. These results must be compared to those obtained with other type of preliminary screening conducted for longer time on plants grown on soil or inert material in pot systems to complete a preliminary scheme of crops phenotypic characterization. In particular, here we report a preliminary phenotypical characterization of three different cultivars of *L. corniculatus* originating from different European geographic areas using *L. japonicus* plants for comparison. We screened growth capacities and symbiotic performances in the presence and absence of nitrogen and phosphate and/or rhizobia.

## 2. Results 

### 2.1. Experimental Design

**Figure 1:**
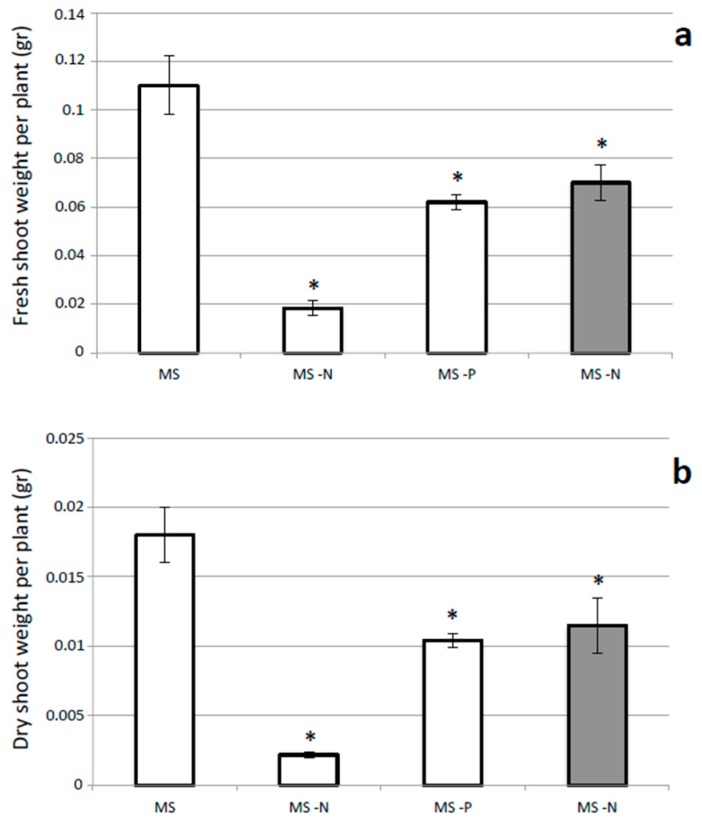
Quantitative analysis of growth parameters of *L. japonicus* plants grown in the presence/absence of N and P sources. (**a**) Fresh shoot weight per plant; (**b**) Dry shoot weight per plant. The different N and P regimes are indicated and grey bars represent values obtained after *M. loti* inoculation. Data bars represent means and SD of measures from three experiments (15 plants per experiment per condition). Data are scored 25 days after sowing (21 days after transferring the plants from H_2_O agar). Asterisks indicate significant differences with values scored on MS medium (*p* < 0.05).

We first used *L. japonicus* plants to check how the presence or absence of N and P sources in Murashige and Skoog (MS) derived media could affect growth capacities, allowing a quantitative phenotypic characterization in our in vitro conditions. In MS complete medium N inorganic nutrients are represented by 19 mM KNO_3_ and 20 mM NH_4_NO_3_, whereas P is present as 1.25 mM KH_2_PO_4_ and these nutrients are omitted in MS derivative media. Nodulation capacity after *M. loti* inoculation is tested in N free conditions. Five days after sowing, seedlings germinated on H_2_O agar plates are transferred on the different media and phenotypical growth parameters; shoot fresh/dry weight, analyzed after 21 days. Germination on H_2_O agar plates allows discarding of unsynchronized seedlings ensuring homogeneous material at the starting of the test. The same timing is maintained for the analysis of the *M. loti* inoculated plants. As expected the absence of N is a more severe limiting conditions for plant growth as compared to P starvation and shoot fresh and dry weights are about 13% of the ones of plants grown in MS control conditions (Figure 1a,b). In addition the absence of P is also significantly affecting the plant growth capacity that drops of about 45% compared to control (Figure 1a,b). The symbiotic interaction in N free medium is sufficient to re-acquire about 60% of the fresh and dry shoot weight of control plants (Figure 1a,b).

### 2.2. Growth Responses of L. corniculatus cvs. to N and P Starvation

**Figure 2:**
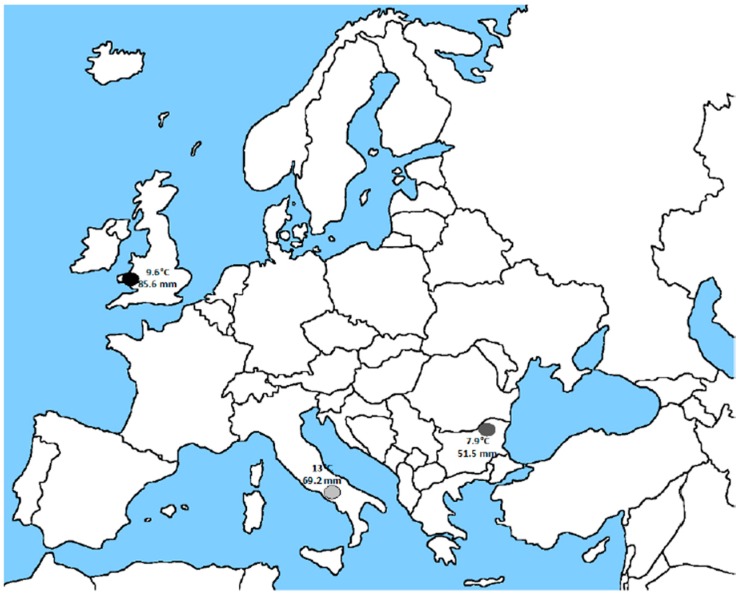
Sites of sampling of the selected *L. corniculatus* cvs. Light grey oval, Canetra cv; dark grey oval Leo cv; Black oval, Targovishte 1 cv. Numbers indicate annual average temperature and rainfall of the sampling sites.

The same media composition utilized to highlight *L. japonicus* growth phenotypes in the presence of different concentration of N and P sources without *M. loti* inoculation has been used to characterize different *Lotus corniculatus* cvs originated from completely different European areas. The three selected cultivars are: Leo original from Wales; Canetra, a local variety isolated from a region of central Italy; Targovishte 1, isolated from east Bulgaria. These cultivars have been selected because adapted to completely different environmental conditions (temperature, rainfall, soil conditions) where high fitness and productivity parameters may be linked to specific phenotypic traits (Figure 2). *L. corniculatus* Leo cv has been already exploited in mixed pastures for testing hydrocarbon degradation in contaminated soil after inoculation with bacteria degrading strains [27,28] and for assessing herbicide resistance and freezing tolerance through in vitro studies [29,30]. Furthermore, natural and transgenic lines polymorphic for condensed tannins quantity in vegetative tissues have been selected and characterized in the Leo cv [31,32,33,34]. More recently a molecular characterization of the Leo cultivar has been achieved through a genome-wide analysis with identification of salt stress responsive genes, functionally characterized in *Arabidopsis thaliana* [35,36]. Targovishte 1 cv has been recently analyzed for comparison of biochemical composition, enzymatic activities and symbiotic performances in pure and grass mixture pot cultivations, indicating significant improvements of these parameters in combined growth conditions [37,38]. 

**Figure 3:**
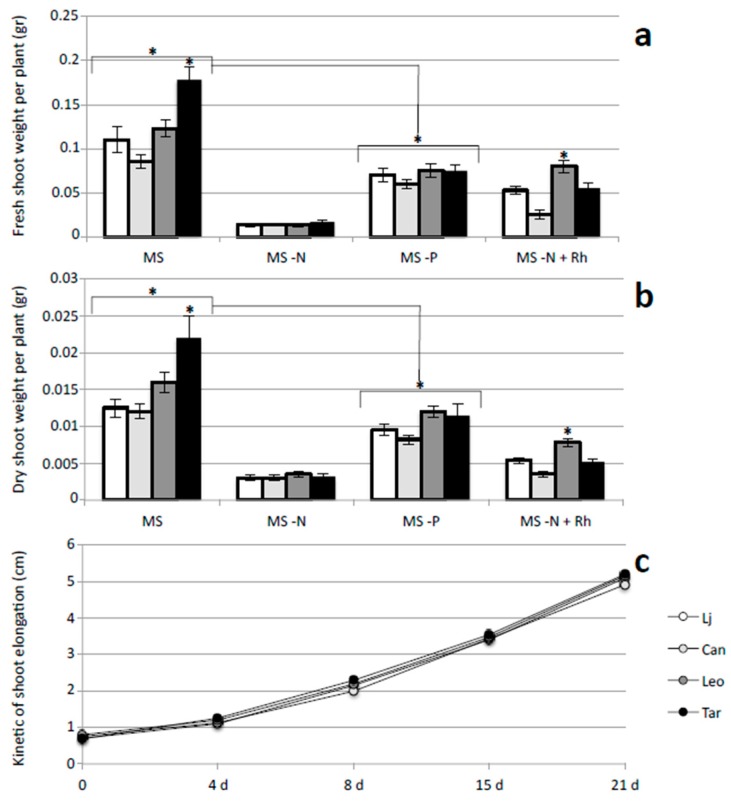
Quantitative analysis of shoot growth parameters of *L. japonicus* and *L. corniculatus* cvs Canetra, Leo and Targovishte 1, grown in the presence/absence of N and P sources in symbiotic and non symbiotic conditions. (**a**) Fresh shoot weight per plant; (**b**) Dry shoot weight per plant; (**c**) Kinetic of shoot elongation on MS complete medium. The different N and P regimes and when performed, *M. loti* inoculation is indicated. In panel (**c**) days after transfer on MS medium are indicated on the X axis; at 0 the sizes of seedlings at the moment of the transfer on MS plates are reported. White bars: *L. japonicus*. Light grey bars: *L. corniculatus* cv Canetra. Dark grey bars: *L. corniculatus* cv Leo. Black bars: *L. corniculatus* cv Targovishte 1. Data bars represent means and SD of measures from three experiments (10 plants per experiment per condition). Data are scored 25 days after sowing (21 days after transferring the plants from H_2_O agar). Asterisks over horizontal lines indicate significant differences for all plants tested in those conditions. Asterisks over single bars indicate significant differences for Targovishte 1 cv and Leo cv plants (*p* < 0.05).

In order to minimize micro-environmental effects due to culturing conditions the different cvs are grown in the same Petri dishes and phenotypes evaluated by using *L. japonicus* plants for comparison of quantitative parameters. The efficacy of the experimental in vitro conditions designed for the phenotypical quantitative screening is confirmed by the observation that all the three *L. corniculatus* cvs tested show a significant decrease of shoot weight growth parameters in the absence of P nutrient in the growth medium (Figure 3a,b). Interestingly, Targovishte 1 cv. shows a better growth capacity in terms of both fresh and dry shoot weight in MS rich medium (about 40% increase), when compared to *L. japonicus*, Canetra and Leo (Figure 3a,b). The same effect is not observed in the absence of N probably because in that case, the severity of the shoot weight phenotype does not allow for quantitating possible effects related to the presence of P into the medium. This growth capacity may be related to a more effective P acquisition capacity as we do not observe significant changes of shoot weights measures between different cvs in the absence of P nutrients but even a N/P combination effect, where N is essential for a better P acquisition, must be taken in account (Figure 3a,b). 

**Figure 4:**
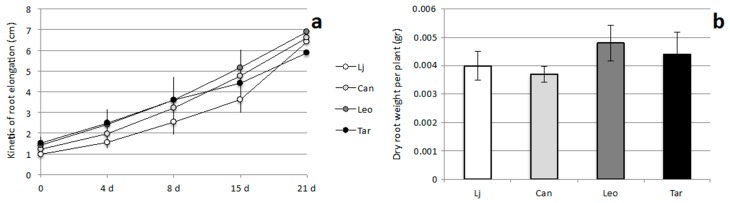
Quantitative analysis of root growth parameters of *L. japonicus* and *L. corniculatus* cvs Canetra, Leo and Targovishte 1, grown on complete MS medium. (**a**) Kinetic of root elongation; (**b**) Dry root weight per plant. In panel (**a**) days after transfer on MS medium are indicated on the X axis; at 0 the sizes of roots at the moment of the transfer on MS plates are reported. Data bars represent means and SD of measures from three experiments (10 plants per experiment per condition).

The agar based experimental set up allows for the following of growth rate parameters of underground and aerial part of the plants within a short period of time. The measures of the kinetic of shoot elongation indicated no differences between cultivars grown on MS medium (Figure 3c). In the same way, the kinetic of primary root elongation did not change significantly on MS medium (Figure 4a) as well as general root architecture (length and number of secondary roots) as indicated by the dry weight values shown in Figure 4b, indicating that a nutrient effect on root developmental program is not responsible for the observed differences in shoot biomass. 

### 2.3. Symbiotic Performances of L. corniculatus cvs. 

**Figure 5:**
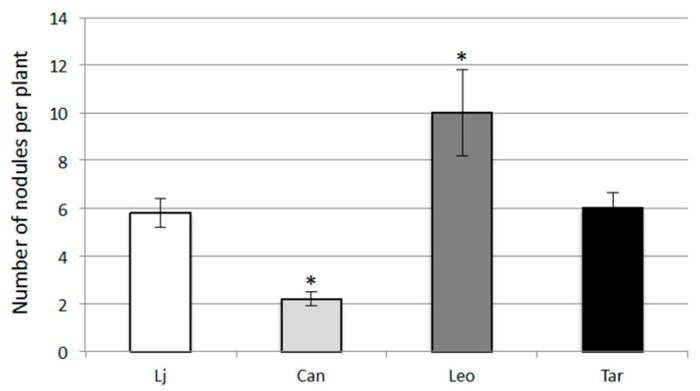
Nodulation capacities of *L. japonicus (Lj)* and *L. corniculatus* cvs. Canetra (Can), Leo and Targovishte 1 (Tar). The plants are grown after inoculation with *M. loti* for three weeks under N-free MS medium. Data bars represent means and SD of measures from three experiments (10 plants per experiment per condition). Data are scored 25 days after sowing (21 days after transferring the plants from H_2_O agar). Asterisks indicate significant differences with nodules scored on *L. japonicus* plants (*p* < 0.05).

The Canetra cv. shows the lowest growth capacities in symbiotic conditions with fresh and dry shoot weights measures fluctuating between 47% and 60% of those detected on other Lotus plants (Figure 3a,b). This low symbiotic performance correlates to the scarce nodulation capacity as indicated by the lower number of nodules formed in Canetra plants when compared to other cvs (Figure 5). On the other hand, Leo cv. shows a clear-cut increased nodulation capacity with higher nodule number in N free conditions when compared to other plants (Figure 5). The increased number of nodules observed on Leo cv. is consistent to the higher shoot growth parameters detected in symbiotic conditions (Figure 3a,b). 

## 3. Discussion

The integration of different legume species into grassland systems is a procedure that requires further investigation before full establishment. Different phenotypic traits can be required for increasing competitiveness in different geographic areas characterized by specific environmental conditions. Native ecotypes may represent a possibility to provide higher fitness and productivity within specific environments but it is unlikely that single species present all the required traits and hence mixtures and more complex pastures could represent a good option. 

The translatability of the plethora of information obtained from model species and in vitro studies to crops, for the improvement of agricultural applications is a central matter of debate in plant science [39]. One of the problems hampering this path is represented by the difficulty to evaluate improved traits in field conditions and one main approach is to study crop performances in conditions that are as close as possible to the field. Pot trials using disturbed/undisturbed soil or inert material have been often used as a preliminary tool in many studies on soil fertility and plant growth [40]. The relatively small volume of soil/inert material in pots compared to the larger rooting volume in the field is convenient for testing effects of nutrients and water components [41,42]. Recently, very expensive image technologies for monitoring plant growth have been established that allow a thorough analysis of canopy development exploiting visible, fluorescent or hyperspectral imaging [43,44]. The monitoring of development of the underground root system of course cannot be followed with the same efficacy, although some technical solutions have been proposed. The in vitro-based screening procedure described in this article provides information that cannot be easily achieved with other experimental systems, therefore representing an additional phenotyping approach to be combined to pot trials for preliminary crops characterization. The proposed agar-based experimental system can be potentially applied for evaluating the plant root responses to a plethora of environmental cues in an in vitro strictly controlled system hence representing a possible short-cut system for a preliminary screening. Although one main limitation due to axenic conditions is the short period of plant growth analysis, the agar-based test can provides important information on the immediate effect of different nutritional conditions by following the kinetic of early root system development after consuming of seed nutritional reserves, a crucial step also for the successful establishment of crop cultivations in field. The achievement of this crucial information is also based on the possibility to select synchronized standard seedlings for the analyses, after germination on agar plates. Standard material is extremely important for the phenotypic screening to avoid assessment artefacts, caused by differences in plant size and in particular in primary root size at the start of the test. Such synchronization cannot be achieved with the same uniformity either by direct seeds sowing in pots or by seedlings dwelling in soil or inert material. 

In this article, we analysed two crucial traits for the successful establishment of legume-based mixed pastures, such as phosphate utilization and nodulation capacity in different *L. corniculatus* cultivars (Figure 3, Figure 4 and Figure 5). We focused our attention on *Lotus corniculatus*, legume specie amenable to classical in vitro growth conditions, characterized by large genetic variability and extensively used as forage for its high content of condensed tannins and intrinsic resilience to unfavourable environmental conditions [21,22,23]. Pasture legumes have a relatively high requirement for phosphorous and normally adequate application of phosphate fertilizer is a primary requirement for legumes to succeed. In addition to limiting plant growth, phosphorous starvation markedly impairs persistence of pasture legumes by negatively affecting different steps of symbiotic interaction such as nodule initiation, development and functioning [45,46]. In particular, P deficiency seems to affect carbon sources allocation to nodules in N_2_ fixing legumes [47]. Our phenotypic characterization identified Targovishte 1 as a cultivar with improved growth capacity in a standard MS medium composition. This phenotypic trait is not observed when plants are grown in the same media without phosphate (Figure 3a,b) suggesting an improved acquisition of this nutrient compared to other Lotus plants. An increased phosphorus absorption per unit of root biomass has been already reported for *Lupines* spp., *Lotus pedunculatus*, *Lotus angustissimus* and *Lotus corniculatus* and this feature seems to be crucial for improving legumes competitiveness in grassland systems [48,49]. Interestingly, high phosphorus use efficiency for plant biomass accumulation has been observed in pot trials experiments conducted with Targovishte 1 plants, confirming that the phenotypic trait observed in Figure 3 could reflect a physiological feature of the Bulgarian cultivar [38]. Although we cannot exclude that a variable root touching on the agar substrate may affect biomass formation, we paid particular attention to this point by adjusting careful plant roots during the test. However, such an effect should randomly influence root and shoot elongation while a constant kinetic is maintained between different cultivars in our conditions (Figure 3c and Figure 4a). The second phenotypic treat we analyzed is the nodulation capacity in N starvation conditions. Leo cv shows a clear-cut higher nodule number (almost doubled) compared to other cultivars and *L. japonicus* as well (Figure 5). The molecular basis of the increased number of nodule observed on Leo cv can be different and further investigation is needed to explain this symbiotic phenotype. However, we cannot exclude an effect related to the agar plates-based experimental design, where plant roots cannot be maintained on a complete darkness, so that different ethylene sensitivity of the analyzed cultivars could be responsible of the nodule formation phenotype. Interestingly, this is associated to an increased shoot growth capacity in symbiotic conditions, which does not represent an obvious combination of phenotypic traits. In fact, nitrogen-fixing root nodules are the organs where atmospheric N reduction and release take place, but at the same time these are optional C sink organs that need to assimilate large amount of energy source in the form of photosynthate products either for the formation of nodule primordium [50], and to provide energy for the N fixation performed by the *Rhizobium* and assimilation of the produced ammonium and starch biosynthesis [51,52]. Therefore a very fine regulatory circuit determines the number of nodules sufficient to satisfy N demand but still convenient in terms of energy expenses. Further investigations are needed to evaluate nodulation capacity at different concentration of inorganic and organic N nutrients that could reproduce typical field conditions. 

The phenotypic in vitro characterization presented in this paper highlighted significant differences in terms of growth performances among *L. corniculatus* cvs grown in different N and P conditions. The proposed experimental agar-based scheme can be potentially applied for evaluating the plant responses to many environmental cues hence representing a possible short-cut system to be combined to pot trials for preliminary phenotypic characterization in crops. 

## 4. Experimental Section

### 4.1. Plant Material and Growth Conditions

The *Lotus japonicus* plants used in the phenotypical analyses are ecotype B-129 F12 GIFU. The *Lotus corniculatus* cvs. are: Canetra, Leo and Targovishte 1.

Sterilized seeds are sown on H_2_O agar plates and left over night at 4 °C cap side down. After 24 h in the dark in the growth chamber, Petri dishes are exposed to light and kept in a vertical position. Care is taken to maintain the young emerging roots in contact with the solid substrate [53]. Plants are cultivated in a growth chamber with a light intensity of 200 μmol m^−2^ s^−1^ at 23 °C with a 16 h/8 h day/night cycle. To minimize random effects related to different micro-environmental conditions associated to positions inside the growth chamber, the different cultivars are grown in the same Petri dish (8 plants per Petri dish). Solid growth substrate had the composition of Murashige and Skoog medium (MS), except that, when needed, NH_4_NO_3_ and KNO_3_ or KH_2_PO_4_ are omitted. KCl is added to the medium to replace the potassium source. The media containing vitamins (Duchefa catalogue G0415) are buffered with 2.5 mM 2-(*N*-Morpholino)-Ethanesulfonic Acid (MES; Duchefa, M1503.0250) and pH is adjusted to 5.7 with KOH. 

### 4.2. Inoculation Procedure and Nodulation Test

The inoculation is performed four days after sowing on seedlings with a 1 cm long primary root. Unsynchronized seedlings are discarded at this stage. Plants are grown on sterile filter paper laid on MS derivative solid medium (N free conditions) to obtain a complete and uniform diffusion of the bacteria drop suspension used for inoculation. Each primary root meristem is inoculated with 20 μL of freshly prepared bacterial suspension (10^7^ cells per primary root tip). The strain used for inoculation is *M. loti* R7A grown to mid-log phase in liquid TYR medium supplemented with rifampicin (20 mg/L). Four days after infection, filter paper is removed with forceps and plants are left for on the same Petri dishes. Aluminium foil is wrapped around the lower part of Petri dishes to keep roots in the dark. Nodules are counted and analyzed at 3 weeks post inoculation.

### 4.3. Root Analyses

Primary and secondary roots have been measured with ImageJ software [54].

### 4.4. Statistical Analysis

Statistical analyses have been performed using the VassarStats ANOVA program available at: http://faculty.vassar.edu/lowry/VassarStats.html.

## References

[1] Graham P.H., Vance C.P. (2003). Legumes: Importance and constraints to greater use. Plant Physiol..

[2] Kinkema M., Scott P.T., Gresshoff P.M. (2006). Legume nodulation: Successful symbiosis through short- and long-distance signalling. Funct. Plant Biol..

[3] Rochon J.J., Doyle C.J., Greef J.M., Hopkins A., Molle G., Sitzia M., Scholefield D., Smith C.J. (2004). Grazing legumes in Europe: A review of their status, management, benefits, research needs and future prospects. Grass Forage Sci..

[4] Luscher A., Mueller-Harvey J.F., Soussana R.M., Peyraud J.L. (2014). Potential of legume-based grassland-livestock systems in Europe: A review. Grass Forage Sci..

[5] Kusvuran A., Ralice Y., Saglamtimur T. (2014). Determining the biomass production capacities of certain forage grasses and legumes and their mixtures under Mediterranean regional conditions. Acta Adv. Agric. Sci..

[6] Pypers P.S., Verstraete C.P., Merckx R. (2005). Changes in mineral nitrogen, phosphorus availability and salt-extractable aluminium following the application of green manure residues in two weathered soils of south Vietnam. Soil Biol. Biochem..

[7] Vanlawe B.J., Wendt K.E., Giller M., Corbeels B., Nolte C. (2014). A fourth principle is required to define conservation agriculture in sub-Saharan Africa: The appropriate use of fertilizer to enhance crop productivity. Field Crops Res..

[8] Caporali F., Campiglia E., Mancinelli R., Paolini R. (2004). Maize performances as influenced by winter cover crop green manuring. Ital. J. Agron..

[9] Carroll B., Gresshoff P.M. (1983). Nitrate inhibition of nodulation and nitrogen fixation in white clover. Z. Pflanzenphysiol..

[10] Day D.A., Carroll B.J., Delves A.C., Gresshoff P.M. (1989). Relationship between autoregulation and nitrate inhibition of nodulation in soybeans. Physiol. Plant..

[11] Fujikake H., Yamazaki A., Ohtake N., Sueyoshi K., Matsuhashi S., Ito T., Mizuniwa C., Kume T., Hashimoto S., Ishioka N.S. (2003). Quick and reversible inhibition of soybean root nodule growth by nitrate involves a decrease in sucrose supply to nodules. J. Exp. Bot..

[12] Barbulova A., Rogato A., D’Apuzzo E., Omrane S., Chiurazzi M. (2007). Differential effects of combined N sources on early steps of the Nod factor-dependent transduction pathway in *Lotus japonicus*. Mol. Plant-Microbes Interact..

[13] Omrane S., Ferrarini A., D’Apuzzo E., Rogato A., Delledonne M., Chiurazzi M. (2009). Symbiotic competence in *Lotus japonicus* is affected by plant nitrogen status: Transcriptomic identification of genes affected by a new signalling pathway. New Phytol..

[14] Omrane S., Chiurazzi M. (2009). A variety of regulatory mechanisms are involved in the nitrogen-dependent modulation of the nodule organogenesis program in legume roots. Plant Signal Behav..

[15] Okamoto S., Ohnishi E., Sato S., Takahashi H., Nakazono M., Tabata S., Kawaguchi M. (2009). Nod factor/nitrate-induced CLE genes that drive HAR1-mediated systemic regulation of nodulation. Plant Cell Physiol..

[16] Reid D.E., Ferguson B.J., Gresshoff P.M. (2011). Inoculation- and nitrate-induced CLE peptides of soybean control NARK-dependent nodule formation. Mol. Plant Microbe Interact..

[17] Jeudy C., Ruffell S., Freixes S., Tillard P., Santoni A.L., Morel S., Journet E.P., Duc G., Gojon A., Lepetit M. (2010). Adaptation of *Medicago truncatula* to nitrogen limitation is modulated via local and systemic nodule developmental responses. New Phytol..

[18] Ledgard S.F., Steele K.W. (1992). Biological nitrogen fixation in mixed legume/grass pastures. Plant Soil..

[19] Vasileva V., Ilieva A. (2016). Sustainable yield index in some mixtures. J. Glob. Innov. Agric. Soc. Sci..

[20] Waghorn G., Ulyatt M., John A., Fisher M. (1987). The effect of condensed tannins on the site of digestion of amino acids and other nutrients in sheep fed on *Lotus corniculatus* L.. Br. J. Nutr..

[21] Jean-Blain C. (1998). Nutritional and toxicological aspects of tannins. Rev. Med. Vet..

[22] Banuelos G., Cardon G., Mackey B., Ben-Asher J., Wu L., Beuselink P. (1992). Boron and selenium removal in boron-laden soil by birdsfoot trefoil. Lotus Newsl..

[23] Blumenthal M., McGraw R., Beuselink P. (1999). Lotus adaptation, use and management. Trefoil: The Science and Technology of Lotus.

[24] Weeb K., Jones S., Robbins M., Minchin F. (1990). Characterization of transgenic root culture of *Trifolium repens*, *T. pratense* and *Lotus corniculatus* and transgenic plants of *L. corniculatus*. Plant Sci..

[25] Handberg K., Stougaard J. (1992). *Lotus japonicus*, an autogamous, diploid legume species for classical and molecular genetics. Plant J..

[26] Jiang Q., Gresshoff P.M. (1997). Classical and molecular genetics of the model legume *Lotus japonicus*. Mol. Plant-Microbes Interact..

[27] Yousaf S., Andria V., Reichenauer T.G., Smalla K., Sessitsch A. (2010). Phylogenetic and functional diversity of alkane degrading bacteria associated with Italian ryegrass (*Lolium multiflorum*) and Birdsfoot trefoil (*Lotus corniculatus*) in a petroleum oil-contaminated environment. J. Hazard. Mater..

[28] Yousaf S., Ripka K., Reichenauer T.G., Andria V., Azfal M., Sessitsch A. (2010). Hydrocarbon degradation and plant colonization by selected bacterial strains isolated from Italian ryegrass and birdsfoot trefoil. J. Appl. Microbiol..

[29] Keith C.N., McKersie B.D. (1986). The effect of abscisic acid on the freezing tolerance of callus cultures of *Lotus corniculatus*. Plant Physiol..

[30] Pofelis S., Le H., Grant W.F. (1992). The development of sulfonylurea herbicide-resistant birdsfoot trefoil (*Lotus corniculatus*) plants from in vitro selection. Theor. Appl. Genet..

[31] Carron T.R., Robbins M.P., Morris P. (1994). Genetic modification of condensed tannins biosynthesis in *Lotus corniculatus*. 1. heterologous antisense dihydroflavonol reductase down-regulates tannin accumulation in “hairy root” cultures. Theor. Appl. Genet..

[32] Robbins M.P., Paolocci F., Hughes J.W., Turchetti V., Allison G., Arcioni S., Morris P., Damiani F. (2003). *Sn*, a maize bHLH gene, modulates anthocyanin and condensed tannin pathways in *Lotus corniculatus*. J. Exp. Bot..

[33] Paolocci F., Bovone T., Tosti N., Arcioni S., Damiani F. (2005). Light and an exogenous transcription factor qualitatively and quantitatively affect the biosynthetic pathway of condensed tannins in *Lotus corniculatus* leaves. J. Exp. Bot..

[34] Paolocci F., Robbins M.P., Madeo L., Arcioni S., Martens S., Damiani F. (2007). Ectopic expression of a basic helix-loop-helix gene transactivates parallel pathways of proanthocyanidin biosynthesis. structure, expression analysis, and genetic control of leucoanthocyanidin 4-reductase and anthocyanin reductase genes in *Lotus corniculatus*. Plant Physiol..

[35] Sun Z.M., Zhou M.L., Xiao X.G., Tang Y.X., Wu Y.M. (2014). Genome-wide analysis of AP2/ERF family genes from *Lotus corniculatus* shows LcERF054 enhances salt tolerance. Funct. Integr. Genom..

[36] Sun Z.M., Zhou M.L., Wang D., Tang Y.X., Lin M., Wu Y.M. (2016). Overexpression of the *Lotus corniculatus* soloist gene LcAP2/ERF107 enhances tolerance to salt stress. Protein Pept. Lett..

[37] Ilieva A., Vasileva V., Katova A. (2015). The effect of mixed planting of birdfoot trefoil, sainfoin, subterranean clover and tall fescue on nodulation and nitrate reductase activity in shoots. J. Glob. Agric. Ecol..

[38] Ilieva A., Vasileva V. (2016). Biochemical composition and phosphorus use efficiency in some mixtures. Intern. J. Bioassays.

[39] Nelissen H., Moloney M., Inzè D. (2014). Translational research: From pot to plot. Plant Biotechnol. J..

[40] Ogunkule A.O., Beckett P.H.T. (1988). The efficiency of pot trials, or trials on undisturbed soil cores, as predictors of crop behaviour in the field. Plant Soil.

[41] Sherrell C.G., Saunders W.M.H. (1974). Factors affecting growth and response of white clover in pots to appled phosphorus: I level of watering at which pots are maintained. N. Z. J. Agric. Res..

[42] Stevenson D.S. (1978). Interaction between soil volume and added fertilizers under continuous watering. Can. J. Sci..

[43] Dhondt S., Wuyts N., Inzè D. (2013). Cell to whole-plant phenotyping: The best is yet to come. Trends Plant Sci..

[44] Fiorani F., Schurr U. (2013). Future scenarios for plant phenotyping. Annu. Rev. Plant Biol..

[45] Israel D.W. (1987). Investigation of the role of phosphorous in symbiotic dinitrogen fixation. Plant Physiol..

[46] Vance C.P., Uhde-Stone C., Allan D.L. (2003). Phosphorous acquisition and use: Critical adaptations by plants for securing a non-renewable resource. New Phytol..

[47] Kleinert A., Venter M., Kossmann J., Valentine A. (2014). The reallocation of carbon in P deficient lupins affects biological nitrogen fixation. J. Plant Physiol..

[48] Schwendiman J.L., Foster R.B., Haglund O.K. The influence of climate, soils and management on the root development of grass species in western states. Proceedings of the Annual Meeting American Forage and Grassland Council.

[49] Torales A.T.A., Deregibus V.A., Moauro P.R. (1998). Phosphorous absorption capacity of *Lotus corniculatus* and Festuca arundinacea during sward establishment. N. Z. J. Agric. Res..

[50] Complainville A., Brocard L., Roberts I., Dax E., Sever N., Sauer N., Kondorosi A., Wolf S., Oparka K., Crespi M. (2003). Nodule initiation involves the creation of a new symplasmic field in specific root cells of medicago species. Plant Cell.

[51] Vance C.P., Dilworth M.J., James E.K., Sprent J.I. (2008). Carbon and Nitrogen metabolism in legume nodules. Nitrogen-Fixing Leguminous Symbioses.

[52] D’Apuzzo E., Valkov T.V., Parlati A., Omrane S., Barbulova A., Sainz M.M., Lentini M., Esposito S., Rogato A., Chiurazzi M. (2014). PII overexpression in *Lotus japonicus* affects nodule activity in permissive low nitrogen conditions and increases nodule numbers in high nitrogen treated plants. Mol. Plant-Microbes Interact..

[53] Barbulova A., D’Apuzzo E., Rogato A., Chiurazzi M. (2005). Improved procedures for in vitro regeneration and for phenotypical analysis in the model legume *Lotus japonicus*. Func. Plant Biol..

[54] Schneider C.A., Rasband W.S., Eliceiri K.W. (2012). NIH Image to ImageJ: 25 years of image analysis. Nat. Methods.

